# “Eating isn’t just about paying attention—It's about the self-regulation of sensory attention while eating!”: Exploring mindful eating by examining sensory attention and non-judgmental awareness in the context of eating cessation

**DOI:** 10.1177/02601060241289508

**Published:** 2024-10-14

**Authors:** Michail Mantzios

**Affiliations:** College of Psychology, 1725 Birmingham City University, Birmingham, UK

**Keywords:** Eating cessation, mindful eating behavior, attentive eating, sensory attention, non-judgmental awareness

## Abstract

**Background:** Recent research has focused on several critical categories linked to eating cessation, including decreased food appeal (DFA), physical satisfaction (PS), planned amount (PA), self-consciousness (SC), and decreased priority of eating (DPE). However, how these factors connect to mindful eating remains unclear. **Aim:** The present study sought to re-examine the relationships between these categories using the Reasons Individuals Stop Eating Questionnaire (RISE-Q) and the Mindful Eating Behavior Scale-Trait (MEBS-T). The MEBS-T measures the self-regulation of sensory attention (SA) while eating, and comprises two subscales—SA and non-judgmental awareness (NJA)—that align with the principles and literature of mindful eating and mindfulness, and create a possible distinction between attentive vs. regulated or reoccurring attentive eating. **Methods:** A sample of 485 participants was recruited, and Pearson's correlations and multiple linear regressions were used to assess the associations between the MEBS-T and RISE-Q variables. **Results:** The results indicated a positive association between mindful eating and DFA, PS, and PA, supported by both the SA and NJA subscales. Additionally, unique relationships were observed between the SA and NJA subscales and the SC and DPE scales of the RISE-Q. Multiple linear regression analyses further confirmed these associations and highlighted an increased variance of NJA. **Conclusion:** The findings emphasize the significance of NJA in understanding the cessation of eating due to negative emotions and social comparison. The study also underlines the potential role of NJA in enhancing attentive eating and its relevance for weight regulation management strategies. Further research is warranted to explore the implications of these findings and their potential applications in improving mindful eating practices.

## Introduction

Research has explored five key categories related to the termination of eating, as identified in the Reasons Individuals Stop Eating Questionnaire (RISE-Q; Cunningham et al., 2021). These categories are decreased food appeal (DFA), physical satisfaction (PS), planned amount (PA), self-consciousness (SC), and decreased priority of eating (DPE), which are part of the RISE-Q (Cunningham et al., 2021), and will be briefly explored next. DFA refers to sensory changes and reduced liking, wanting, or enjoyment of food, leading to consumption cessation (e.g., Hetherington, 1996). PS, on the other hand, involves feeling satisfactorily full, influencing portion selection and eating cessation (e.g., Hinton et al., 2021). PA concerns decisions made before eating about the amount to consume, closely linked to actual consumption (e.g., Brunstrom, 2011; Fay et al., 2011). SC reflects negatively comparing eating behavior to others’ eating, leading to intake suppression (e.g., Herman et al., 2003), while DPE represents decreased motivation or interest in eating (e.g., Adise et al., 2018). Chawner et al.'s (2022) study found PS, decreased food priority, SC, and diminished food appeal were positively associated with satiety responsiveness. Moreover, decreased food priority was found to be negatively correlated with overeating and weight gain. Interestingly, the same research has shown a positive link between PS and sensitivity to internal satiation cues, measured by the Mindful Eating Questionnaire (Chawner et al., 2022). Such findings were consistent with previous studies suggesting that mindful eating can help regulate eating and reduce overeating, as being more attentive to the present moment enhances the perception of satiety (e.g., Mantzios and Wilson, 2015).

However, it is important to note that the Mindfulness Eating Questionnaire (Framson et al., 2009), which was used in Chawner et al.'s work, has faced challenges in serving as a valid psychometric assessment tool (Carrière et al., 2022; Hulbert-Williams et al., 2013; Peitz and Warschburger, 2022; Winkens et al., 2018). Two primary issues have been identified. First, the questionnaire does not align with mindfulness and mindful eating principles. Second, it shows potential inflations in associations, as several of its items do not assess mindful eating, but instead other eating behaviors, such as emotional eating (Mantzios, 2023a). Further explorations with more valid tools are necessary to draw the potential associations between mindful eating and the reasons leading to deliberate eating cessation.

Mantzios (2020) introduced a definition for MEB, which is as follows: “the sustained attention to a sensory element of the eating experience (e.g., the taste) and a non-judgmental (or non-evaluative) awareness of thoughts and feelings that are incongruent to the sensory elements of the present eating experience” (p. 369). This definition offers a more robust and precise foundation for research, founded on empirical evidence and the principles of secular mindfulness. Mindfulness is commonly defined as an awareness that emerges by deliberately focusing on the present moment with a non-judgmental attitude (Kabat-Zinn, 1990). A non-judgmental attitude enables the self-regulation of attention in mindfulness practices (Bishop, 2004). The same is true for mindful eating practices, where non-judgmental awareness (NJA) enables reoccurring attention or “*the self-regulation of sensory attention while eating*.” In addition, Mantzios (2023b) introduced the Mindful Eating Behavior Scale-Trait (MEBS-T) and corresponding practices, which are in alignment with both mindful eating and mindfulness theories. The MEBS-T includes two subscales that assess SA and NJA, aligning with the principles found in both mindfulness and mindful eating literature. This scale offers an opportunity to investigate the relationships of *attention* and *non-judgment*, both parts of mindful eating, with the RISE-Q, which can potentially lead to more accurate findings and implications for mindful eating practices. Also, a recent review by Tapper (2017) suggested that there was no conclusive evidence to support the idea that acceptance or non-judgment could lead to changes in eating behavior. Consequently, some researchers (e.g., Fekih-Romdhane et al., 2023; Winkens et al., 2018) chose to focus solely on the attentive aspects of mindful eating (elsewhere identified as “attentive eating,” e.g., Whitelock et al., 2019), rather than attention and non-judgment. The MEBS provides a unique opportunity to explore the potential of attentive practices independently, aiming to verify or challenge previous notions and directives on mindful eating.

For example, the RISE-Q is relatively new (Cunningham et al., 2021) and derived from past research, and theory-driven frameworks that propose specific investigations of satiation dependent on individual differences. Sensory-specific satiety (SSS) is just one component measured in the RISE-Q (i.e., DFA subscale) and is characterized by a decrease in the perceived pleasantness of consumed food (e.g., Cox et al., 2016) which could be an element that is uniquely associated with the *attention* to one's eating, but even more so with the ability to revisit sensory experiences (i.e., NJA). Exploring the differences serves for the advancement of MEB, and a directive in measuring and practicing mindful eating.

The present research aimed to reevaluate findings from a previous study by Chawner et al. (2022) using a validated mindful eating scale. Additionally, the study aimed to explore potential connections between SA, NJA during eating, and the decision to stop eating. The uniqueness of the MEBS-T can shed light on whether SA (as in attentive eating), or SA and the reoccurring of such attention (as in SA and NJA, i.e., mindful eating behavior) contribute to the cessation of eating. Contributing to the differentiation between attentive and mindful eating, with the latter incorporating both SA and NJA may further evidence the potential of interventions in enhancing SSS, and potentially other reasons to cease eating.

## Methods

### Participants

The sample consisted of 485 participants (273 Females), with a mean age of 26.64 (*SD *= 8.98) and a mean BMI of 24.44 (*SD *= 4.92). Approximately half of the participants were White (*n* = 256), followed by smaller groups of Black (*n* = 94), Asian (*n* = 67), and mixed race (*n* = 58) participants (10 participants did not disclose any racial or ethnic background). With significance set at .05, small effect size, power = .80, a sample size of 481 participants was aimed to be recruited (Cohen, 1992; Faul et al., 2009). Exclusion criteria were self-defined current mental health and eating disorder diagnoses, as well as being under the age of 18.

### Materials

Reasons Individuals Stop Eating Questionnaire (RISE-Q-15; Chawner et al., 2022). Originally developed by Cunningham et al. (2021), the RISE-Q has five distinct subscales, each designed to explore various facets influencing why individuals halt their eating. These subscales encompassed “Decreased Food Appeal,” “Physical Satisfaction,” “Planned Amount,” “Self-Consciousness,” and “Decreased Priority of Eating.” The study's results indicated strong internal consistency, as demonstrated by Cronbach's alpha coefficients: *α* = .88 for DFA, *α* = .77 for PS, *α* = .66 for PA, *α* = .82 for SC, and *α* = .75 for decreased food priority. These findings underscore the reliability and robustness of the RISE-Q's subscales in assessing the factors contributing to eating cessation.

MEBS-T (Mantzios, 2023b) contains 8 items that measure two components of mindful eating behavior: *Sensory Attention* (4 items) and *Non-judgmental Awareness* (4 items). Both factors are descriptive and align with mindful eating behavior and secular mindfulness theory. Sample questions include “I fully taste what I am eating” and “I hold my attention on what I am eating, despite recognizing the occurrence of thoughts and/or feelings while I am eating.” The scale utilizes a 4-point Likert scale with responses ranging from 1 (strongly disagree) to 4 (strongly agree). Internal consistency for the total score of the scale was good (*α* = .78), and similarly for the subscales “sensory attention” (*α* = .82) and “non-judgmental awareness” (*α* = .83).

### Procedure

Participants were given access to an online platform (Qualtrics) where they initially reviewed the participant information sheet and consent forms. Once they provided their consent, they were directed to the demographics page and the psychometric materials (i.e., MEBS-T and RISE-Q). For half of the participants, the order of administration of these psychometric tools was reversed to balance the sequence. It is worth noting that the order of administration had no discernible impact on the results. Upon finishing the materials, participants were guided to a debriefing page, marking the conclusion of their participation.

### Data analysis

The data were analyzed through bivariate correlations and multiple linear regressions to discern the extent to which mindful eating subscales could predict the 5 factors influencing individuals’ cessation of eating. To account for multiple testing, the significance threshold was adjusted to .01 (i.e., .05/5) for each analysis. Here, attentive eating was solely represented by the SA subscale, while mindful eating encompassed both SA and NJA. SPSS 28.0 for Windows was utilized to perform the analyses.

## Results

Pearson's correlations were employed to assess the relationships between the measured variables, as depicted in the correlation matrix in Table 1. The results revealed that mindful eating was positively associated with DFA, PS, and PA in the context of eating cessation, which was further corroborated by both the SA and NJA Mindful Eating Behavior Subscales. Notably, the SA and NJA Mindful Eating Behavior Subscales demonstrated distinct associations with the SC and DPE scales of the RISE-Q. Specifically, SA exhibited a negative correlation with both SC and DPE, while NJA demonstrated a positive correlation with both scales.

**Table 1.** Bivariate correlations between MEBS-T and RISE-Q.

	1.	2.	3.	4.	5.	6.	7.	*M*	*SD*
1. MEBS-T								21.32	3.77
2. SA	.767**							12.04	2.25
3. NJA	.817**	.257**						9.27	2.50
4. DFA	.152**	.118**	.123**					11.96	3.25
5. PS	.173**	.145**	.130**	.563**				14.78	3.61
6. PA	.185**	.151**	.144**	.266**	.473**			14.43	3.71
7. SC	.021	−.117*	.140**	.132**	−.156**	−.059		8.46	4.37
8. DPE	−.009	−.168**	.141**	.241**	.009	−.043	.484**	8.39	4.05

*Note:* RISE-Q: decreased food appeal (DFA), physical satisfaction (PS), planned amount (PA), self-consciousness (SC) and decreased priority of eating (DPE). Mindful Eating Behavior Scale – Trait total score (MEBS – T), sensory attention (SA) and non-judgmental awareness (NJA) Mindful Eating Behavior Subscales.

* *p* < .05.

** *p* < .01.

Furthermore, multiple linear regressions with both SA and NJA Subscales of the MEBS-T were used to predict eating cessation, with outcome variables: DFA, PS, PA, SC, and DPE. The findings suggested similarities to the previous correlation matrix, while also indicating a heightened variance of NJA. The results have been detailed in Table 2.

**Table 2.** Multiple linear regression analyses show the predictive relationship between the Mindful Eating Behavior Scale-Trait (MEBS-T) subscales of the Reasons Individuals Stop Eating Questionnaire (RISE-Q).

Variables	*b* (95% CI)	*p*	β
*DV: Decreased Food Appeal (DFA)*			
Sensory Attention (SA)	.133 (.000; .266)	.050	.092
Non-judgmental Awareness (NJA)	.130 (.010; .250)	.034	.099
*DV: Physical Satisfaction (PS)*			
Sensory Attention (SA)	.189 (.042; .336)	.012	.118
Non-judgmental Awareness (NJA)	.146 (.013; .278)	.032	.101
*DV: Planned Amount (PA)*			
Sensory Attention (SA)	.199 (.048; .350)	.**010**	.121
Non-judgmental Awareness (NJA)	.169 (.032; .306)	.016	.113
*DV: Self-Consciousness (SC)*			
Sensory Attention (SA)	−.320 (−.496; −.143)	**<**.**001**	−.165
Non-judgmental Awareness (NJA)	.318 (.159; .478)	**<**.**001**	.181
*DV: Decreased Priority of Eating (DPE)*			
Sensory Attention (SA)	−.393 (−.555; −.231)	**<**.**001**	−.219
Non-judgmental Awareness (NJA)	.318 (.171; .464)	**<**.**001**	.196

*Note: 95% CI = *95% confidence interval, *DV = *dependent variable, *b* = unstandardized coefficient, *β* = standardized coefficient. Effects with *p *< .01 are in **boldface**.

## Discussion

The current study aimed to reevaluate the findings of an earlier investigation by Chawner et al. (2022) using a validated mindful eating scale, the MEBS-T. Additionally, it sought to examine potential associations to SA and NJA during eating, and the decision to stop eating. The unique attributes of the MEBS-T enable a deeper understanding of the roles played by SA and NJA, highlighting potential distinctions between attention (as in attentive eating) and reoccurring attention (as in mindful eating behavior) in influencing the cessation of eating. This differentiation between attentive and mindful eating provides valuable insights into the contribution of different interventions for enhancing satiety and other drivers for discontinuing eating.

The analysis employing Pearson's correlations revealed notable associations among the investigated variables. Mindful eating exhibited a positive correlation with DFA, PS, and PA within the context of cessation of eating. These results were supported by both the SA and NJA Mindful Eating Behavior Subscales. Noteworthy distinctions emerged as the SA and NJA subscales showed unique connections with the SC and DPE scales of the RISE-Q. Specifically, SA demonstrated negative correlations with both SC and DPE, whereas NJA displayed positive correlations with both scales.

Moreover, this study employed multiple linear regression analyses to predict eating cessation, utilizing both the SA and NJA Subscales of the MEBS-T. Outcome variables include DFA, PS, PA, SC, and DPE. The results mirrored those of the earlier correlations, demonstrating an increased variance of NJA. Importantly, a discernible relationship emerged between SA and NJA concerning SC and decreased priority in eating. This relationship signified the cessation of eating due to negative emotions and the comparison with others’ eating patterns, as well as decreased motivation or interest in eating, respectively. These findings bear implications for future research in effective weight regulation management practices, as they indicate a contrary trend in eating cessation.

Further exploration of these findings could be beneficial, with a potential avenue being the consideration of SA as a mechanism for avoiding or escaping self-conscious emotions and associated critical or judgmental evaluations. SA in isolation may facilitate the avoidance, rather than the embracing of thoughts (as in NJA), underscoring the nuanced role it plays in the practice of mindful eating. Future studies should delve into the potential moderating influence of NJA as a factor in enhancing attentive eating, providing a dimension of self- and emotion-regulation not typically addressed in traditional attentive eating literature and practices.

The findings also raise a critical question on the validity of attentive eating practices being characterized and mindful eating in recent literature. The answer to this question lies in understanding the evolving body of literature and research on both mindful and attentive eating, which is being treated as being the same in some research. For example, despite previous theorization exemplifying that attentive eating practices were distinct from mindful eating practices (Robinson et al., 2014), practices that were initially advocated as being “attentive eating” (Robinson et al., 2014) were later advocated in the literature as being “mindful eating” practices (Tapper and Seguias, 2020). It is crucial to acknowledge that assuming these two concepts and their associated practices as identical is a misconception. Attentive eating refers to being mindful of the sensory aspects of eating, such as taste and texture. In contrast, mindful eating involves not only being attentive to such sensory aspects, but also incorporating the element of non-judgment, to create a method of returning one's attention to the meal. Attentive eating is about paying attention to eating, while mindful eating involves the self-regulation of attention during eating, making them significantly different approaches. Mantzios (2020, 2023a) stressed how both elements contribute to mindful eating, and the significance when considering the literature and findings. The current research contributes an additional layer of comprehension by indicating that SA has a predictive value to cease eating, but not of the same magnitude as NJA of one's eating.

The findings have several important implications and by default future research directions. First, while most of the literature has focused on hunger and satiety as key elements around decision-making to support mindful eating practices, it has often overlooked other crucial factors beyond these that may be of more significance. Second, the practice of the self-regulation of SA while eating (i.e., mindful eating behavior) is essential for enhancing SSS (i.e., a decrease in the perceived pleasantness of consumed food) is not particularly significant for attentive eating without the NJA. Third, studying mindful eating as it is intended—incorporating both attention and NJA—offers a promising path for developing research that is both replicable and truly reflective of mindful eating practices. Collectively, these implications highlight several valuable directions for future research. Future studies should explore additional elements that contribute to mindful eating practices, rather than focusing solely on hunger and satiety. This would provide a more comprehensive understanding and inform practices for clinical and non-clinical use. Also, understanding the impact on SSS could offer insights into how mindful eating practices can become more sustainable, where sensory experiences become the central element of focus and decision-making. Last, but not least, to facilitate more consistent and meaningful research in the field, the true nature of mindful eating, incorporating both attention and non-judgment should be incorporated to establish more accurate and replicable frameworks.

While the study provides valuable insights into the relationships between mindful eating and reasons for eating cessation, three limitations should be considered. First, the research relied on self-report measures, which could be susceptible to response bias. Closely related is the use of a mindful eating scale that was not designed to measure attentive eating, and may lack the full spectrum of attentive eating as imagined and seen by experts in attentive eating. Second, the cross-sectional design of the study prevents any assumptions on causal relationships, and the recruitment method proposes future research seeking a more representative sample. Closely aligned is the exclusion of participants with mental health and/or eating disorder diagnoses, which might have led to an underrepresentation of individuals with relevant experiences in clinical populations. Finally, the study did not account for potential confounding variables, such as cultural differences, which could have influenced the observed associations. Mediterranean diets, the association with a variety of foods, and the interplay between SSS, overeating (Rolls and Hetherington, 1989), and mindful eating remains a significant exploration. Further research addressing these limitations is crucial to provide a more comprehensive understanding of the complex dynamics between mindful eating and eating cessation. Researchers and practitioners should consider the potential reinforcing dynamic of mindful eating, but also of motivations to cease eating in creating more effective and sustainable practices for behavior change.

In conclusion, the present research significantly contributes to the understanding of the intricate relationship between mindful eating and the factors influencing eating cessation. The utilization of the validated MEBS-T provided a valuable mechanism for differentiating the contributing variance of both sensory awareness and NJA for eating cessation, ultimately elucidating distinctions between attentive (i.e., attention) and mindful eating (i.e., reoccurring attention or the self-regulation of attention) and their respective impacts on eating behavior. The results underscore the critical role played by both SA and NJA in shaping individuals’ decisions to cease eating. While the study offers a comprehensive perspective, future research should address identified limitations, consider broader population samples, incorporate self- and emotion-regulation and other SC-related considerations, and adopt experimental methodologies to strengthen causal inferences. These concerted efforts are pivotal in fostering the development of effective strategies that align with healthy eating practices and promote mindful eating behaviors predictive of eating cessation.
